# Exploring Barriers to Mental Health Services Utilization at Kabutare District Hospital of Rwanda: Perspectives From Patients

**DOI:** 10.3389/fpsyg.2021.638377

**Published:** 2021-03-22

**Authors:** Oliviette Muhorakeye, Emmanuel Biracyaza

**Affiliations:** ^1^Department of Clinical Psychology, School of Medicine and Pharmacy, University of Rwanda, Butare, Rwanda; ^2^Department of Community Health, School of Public Health, University of Rwanda, Butare, Rwanda; ^3^Sociotherapy Programme, Prison Fellowship Rwanda (PFR), Member of Prison Fellowship International, Kigali, Rwanda

**Keywords:** barriers, mental health, service utilization, accessibility, awareness, hospital

## Abstract

Barriers to mental health interventions globally remain a health concern; however, these are more prominent in low- and middle-income countries (LMICs). The barriers to accessibility include stigmatization, financial strain, acceptability, poor awareness, and sociocultural and religious influences. Exploring the barriers to the utilization of mental health services might contribute to mitigating them. Hence, this research aims to investigate these barriers to mental health service utilization in depth at the Kabutare District Hospital of the Southern Province of Rwanda. The qualitative approach was adopted with a cross-sectional study design. The participants were patients with mental illnesses seeking mental health services at the hospital. Ten interviews were conducted in the local language, recorded, and transcribed verbatim and translated by the researchers. Thematic analysis was applied to analyze the data collected. The results revealed that the most common barriers are fear of stigmatization, lack of awareness of mental health services, sociocultural scarcity, scarcity of financial support, and lack of geographical accessibility, which limit the patients to utilize mental health services. Furthermore, it was revealed that rural gossip networks and social visibility within the communities compounded the stigma and social exclusion for patients with mental health conditions. Stigmatization should be reduced among the community members for increasing their empathy. Then, the awareness of mental disorders needs to be improved. Further research in Rwanda on the factors associated with low compliance to mental health services with greater focus on the community level is recommended.

## Introduction

Mental disorders constitute a mammoth global burden (Saxena et al., 2007; Kessler et al., 2009). Lack of access to and utilization of mental health services remains particularly prominent in the low- and middle-income countries (LMICs) (Demyttenaere et al., 2004; Jack-Ide and Uys, 2013; Ali and Agyapong, 2020). The World Health Organization (WHO) states that mental health is the state of well-being in which people realize their abilities that help them to cope with normal stresses of life, work productively, and contribute to their community welfare and resilience (WHO, 2004; Saxena et al., 2007; World Health Organization, 2015). Thus, mental health is an integral part of health including different aspects of activities that are directly and indirectly related to promotion of mental well-being, prevention and treatment of mental disorders, and rehabilitation of mentally disordered people (WHO, 2003; Glderisi et al., 2010). Although it is known that mental disorders are among the major burdens that lead to disabilities with the estimated prevalence of 37% of all healthy life years lost through mental disorders (Mojtabai et al., 2010; Holden et al., 2013), it was recommended that mental health services should be made available at all potential levels, mainly in the communities and in medical settings. Despite an increase in mental disorders, earlier studies documented that only 32% of people worldwide utilize community intervention facilities, and this includes any type of care to patients with mental disorders seeking mental health interventions outside of the hospitals. While earlier studies indicated that in several Arabic countries, the budget for mental healthcare services is not allocated (Rao et al., 2007), preceding studies indicated that 30% of countries worldwide do not have a budget for mental health at all. This problem has been found in sub-Saharan African countries, such as in Uganda where the national budget spent on mental health services remained very low (Raja et al., 2010).

Mental disorders continue to increase, and these disorders remain poorly understood mostly in developing countries (Wittchen et al., 2003; Wainberg et al., 2017; Alloh et al., 2018). Preceding studies found that mental disorders are less prioritized in most of developing countries in terms of policies, health services, and research, and only 36% of people with mental disorders are covered in developing countries while >92% of people are provided mental health services in developed countries (Glderisi et al., 2010; Abdelgadir, 2012). In some countries, mental health services and resources are available but they are not accessed as it could be due to different factors such as stigma, poverty, and discrimination toward patients with mental health conditions. This discrepancy may affect the access to mental health services and may worsen mental health disorders of the patients (WHO, 2001, 2019; Abdelgadir, 2012; Thyloth and Singh, 2016). Recent studies obviously indicated multiple barriers to use and the utilization of mental health services in developing countries where three-quarters of the patients with mental disorders do not have access to mental health services (Thyloth and Singh, 2016; Ali and Agyapong, 2020). They revealed that the treatment gap for mental health in developing countries is higher than in developed counties (WHO, 2002; Wainberg et al., 2017). Prior studies indicated that the major barriers to mental healthcare access comprise limited availability and affordability of mental healthcare services, insufficient mental healthcare strategies, lack of education about mental disorders, negative attitudes toward mentally disordered patients, and stigma (Saxena et al., 2007; Ayele et al., 2011; Moses et al., 2011).

Moreover, preceding studies documented that the barriers to the utilization of mental health services included shame and stigma of being diagnosed with mental disorders, sociocultural influences, social marginalization for the persons with mental illness, less prioritization of mental healthcare services, scarcity of human and financial resources, physical and psychosocial violence experienced by patients with mental disorders, difficulty of access to geographical areas where the patients reside, and difficulty in charging poorly organized services (WHO, 2002; Glderisi et al., 2010; Abdelgadir, 2012; Jacob and Patel, 2014; Atilola, 2015; Vigo et al., 2018). Studies in sub-Saharan African countries revealed that barriers such as financial means scarcity, poor awareness of the mental disorder, poor knowledge of mental health services, poor quality of services, negative beliefs about healthcare provision, and sociocultural and physical barriers hinder the utilization of the mental health services (Henderson et al., 2013; Jack-Ide and Uys, 2013; Tumbwene et al., 2015; Mugisha et al., 2019). Studies in Kenya established that patients with mental disorders mistrust the healthcare providers, experience barriers like stigma related to their mental disorder, experience sociocultural misunderstandings, resist to therapies, and experience barriers to medical infrastructures resulting in their difficulty in access to and utilization of mental health services (Musyimi et al., 2017). Earlier studies in Uganda, Kenya, and Tanzania discovered that the other important factors that hamper the adherence to mental health interventions include poor accessibility of clinical guidelines to provide mental health services and obstructions due to a limited number of trained mental healthcare providers (Henderson et al., 2013; Atilola, 2015; Mugisha et al., 2019). Another challenge is that there are several mentally ill patients who seek mental health support interventions from traditional healers and faith healers instead of accessing mental health interventions from trained mental health providers in medical or psychiatric settings (Saraceno et al., 2007; Barrow, 2016). A research carried out in Ethiopia revealed that the main impediments to non-adherence to mental health services are that the mentally ill people denied taking mental health services because of thinking that they would get better later and that they also want to solve their mental problems without seeking health care from mental health providers; lack of medical infrastructures; negative attitudes of healthcare providers toward mentally ill patients; and preference to get alternative forms of mental health services (Wakida et al., 2018; Negash et al., 2020). So, mental disorders affect hundreds of millions of people and if left untreated create enormous suffering, disability, and economic loss (Moses et al., 2011; Ambikile and Iseselo, 2017). Further, prevailing public-health priority agenda and its effect on funding were the other barriers that weaken the mental health services. Thus, in such context, the complexity of and resistance to decentralization of mental health services; challenges to implementation of mental health care in primary care settings; insufficient number of trained mental health providers; and the frequent scarcity of public-health perspectives in mental health leadership are among the foremost predictors of poor utilization of mental health services (Mojtabai et al., 2010; Abdelgadir, 2012; Molodynski et al., 2017).

The 1994 genocide against the Tutsi in Rwanda killed more than a million people in only a short time (Barrow, 2016) and has affected nearly all Rwandans, and its effects still occur as one of the factors of a higher rate of mental disorders (Saraceno et al., 2007). However, although the government of Rwanda has made efforts to combat with mental disorders associated with this hazardous event through availing financial and human resources for proving mental healthcare services appropriately, the provision of efficient and effective mental health services remains a challenge (Berckmoes et al., 2017; Negash et al., 2020). Later, preceding studies found a remarkable improvement in providing mental health services due to an increase in financial and human resources and increase in the quality of the services provided (Mutuyimana et al., 2019). Evidently, main challenges faced by staff in caring patients with mental disorders included the lack of compliance to medical prescriptions, patients not respecting the appointments, difficulties related to families not supporting their patients, high costs of medications, poor affordability, stigmatization, and difficulties related to families not collaborating with patients' caregivers. Actually, in some health facilities in Rwanda, there is an insufficient number of trained mental health professionals and people with mental disorders have inadequate access to mental healthcare. This may result in negative reception of mental health care (Munyandamutsa et al., 2012; Mutabaruka et al., 2012). Furthermore, access to mental health services in Rwanda remains low, regardless of the efforts the government of Rwanda has made for attenuating the high prevalence of mental disorders (Wakida et al., 2018; Mukamana et al., 2019). To our knowledge, no study was carried out to explore the barriers to the utilization of mental health services from the perspectives of mentally ill patients; this study in part pursues to address this research gap. Given the aforesaid backgrounds based on the erstwhile literature, this study investigated the barriers to access and utilization of mental health services in-depth at the Kabutare Hospital, in the Southern Province of Rwanda, and the results are crucial to designing the strategies to curtail barriers to mental health service utilization, thereby expanding mental health services provided in Kabutare Hospital in Rwanda.

## Methods

### Study Design

The qualitative study approach was adopted with a cross-sectional design among the patients seeking mental health services at the Department of Mental Health of Kabutare Hospital located in the Southern Province, Rwanda. This study design applied thematic analysis to explore the barriers to the access and utilization of mental health services. This qualitative method consists of scientific inquiry applied to collect and work with no numerical data and seek to interpret meaning from the perspectives of the study participants, helping to understand their social life or phenomena through them (Mwaambi, 2017).

### Study Setting and Participants

Kabutare District Hospital is a district hospital that is situated in Huye District, Southern Province of Rwanda. This health facility was established in 1957 as a center for application for medical students. It was built as a section for medical assistants for these students, who studied at Groupe Scolaire de Butare, under the initiative of Brothers of Charity, but later, it officially became a district hospital from 1982, after the beginning of the Brothers of Charity in 1994, and it has become a public hospital. Kabutare Hospital serves the entire population of Huye District which consists of 328,605 inhabitants in an area of 581.5 km^2^. Its density is 510.3 inhabitants per km^2^. This health facility has all the services of a district hospital except ophthalmology service.

The population of our research was the patients with mental disorders at the Mental Health Department of Kabutare District Hospital. The study included 10 subjects who were aged 18–59 years. The average age was 41 years. All the research participants were recruited from the Department of Mental Health of the recruited medical setting. All recruited participants were Rwandans. All the patients who did not fit the inclusion criteria were excluded from the current study. Among the patients who were excluded were those who were not able to communicate verbally. Additionally, patients who did not receive mental health services for at least >1 month were excluded. The research participants were initially recruited by convenience sampling technique. This approach was used for the patients who came to seek for mental health services. The patients who voluntarily agreed to participate in the study were interviewed. So, they willingly partook due to their availability (Mwaambi, 2017).

### Data Collection

Data collection was conducted using the local language “Kinyarwanda” of the eligible participants at the hospital premises using in-depth interviews (IDIs). The venue for data collection was carefully and appropriately chosen to ensure that it is a comfortable place with no distractions for both researchers and interviews. All recruited patients provided information on their experiences and views about barriers to accessibility of mental health care services at the health facility. This important work of collecting data was done from 20th March to 15th April 2019 among 10 patients with mental disorders who were also seeking healthcare services. The interviewed patients were met at the respective offices of the hospital. Among the participants recruited, six were males and four females. Recruited patients had different psychiatric disorders; three patients had anxiety disorders (e.g., posttraumatic stress disorder), two patients were diagnosed with psychosomatic disorders, one patient had psychotic disorder (e.g., schizophrenia), one patient had depression, one patient was epileptic, one patient had substance use disorder (SUD), and one patient had dual diagnosis (a patient with one psychiatric disorder and a SUD). Concerning healthcare services, five participants came to receive chemotherapy medications only, four came for both chemotherapy and counseling, and one came for only counseling. In this research, only two patients denied being interviewed due to their availability that did not allow them to participate in the study. All data about the diagnosis and treatments were obtained from the medical files availed by the psychiatric nurse from the Department of Mental Health at Kabutare Hospital. Indeed, to ensure that the questions in the interview guide were comprehensible, the pilot testing exercise was conducted among three mentally ill patients from Kabutare Hospital before the data collection. This pilot testing resulted in minor corrections of the interview guide especially in the wording of new questions.

Moreover, the researchers did the literature review before and after collecting data for having the wider views from the research participants particularly according to other scholars. Interview guides, attached as appendices, were used for the data collection. Tools such as note taker and tape recorder were used for capturing all possible information that was important in the study. All interviews were digitally recorded in agreements between interviewees and researchers. Initial analysis was carried out manually using the code sheets, which was a thematic approach of the common themes in a data reduction strategy (Power, 2002; Braun and Clarke, 2006; Luborsky and Rubinstein, 2011; Vaismoradi et al., 2013). In addition to that, trustworthiness was importantly used in the current study; hereby, the credibility of the data collected was addressed by using one method of data collection which is IDIs. With this method, data were obtained from patients who came to seek health interventions in the medical setting. The interview guide of this study was used for ensuring consistency during the data collection. Each interview lasted 45–90 min.

### Data Analysis

As the current research is exploratory, data analysis based on the general inductive model resembles anchored theory aiming at giving directions to raw data and making sense to them. After collecting the interviews, all records were transcribed verbatim before the authors performed translation from the local language to English for the purpose of data analysis. All transcriptions and translations were relevantly checked by the authors of this study against the recordings for correctness of information before proceeding to the next set of interviews. To ensure the accuracy of the translated data, the translated transcripts were retranslated back to Kinyarwanda. The whole set of interviews formed a corpus of approximately 27 pages. The entire corpus was translated from the local language of the participants “Kinyarwanda” into “English” by a Rwandan translator who knows the two languages very well and who often helps in the qualitative data gathering in Kabutare District Hospital. The interviews were read in-depth, in Kinyarwanda and English. By utilizing the thematic analysis, the differences and similarities were observed in the text and these were organized into codes, themes, and subthemes.

The interviews were thematically analyzed. The themes of this study were the latent content, mirroring the underlying meaning which cut across data collected from the research participants. All authors who performed thematic analysis read the transcriptions copious times for capturing the sense of the whole and identify the content areas of the transcriptions. The themes were then identified and coded, using a mixture of preset categories and emerging categories. Then, thematic analysis was applied to investigate the barriers to mental health services. All authors analyzed and finalized the analysis together to ensure the objectivity of the results. In this qualitative thematic analysis, the technique of inductive thematic process was employed for linking the themes. This inductive method is an important process of coding the data without trying to fit into a preexisting coding frame or the authors' analytic preconceptions (Nowell et al., 2017). Based on this approach, the researchers generated the themes inductively from the raw data. Subthemes of this study were formed inductively without fitting it into a preexisting coding frame.

### Ethics

Ethical approval was sought from the Institutional Review Board, the College of Medicine and Health Sciences of the University of Rwanda. Before their participation, the participants were explained about the study and then accepted to take part after providing the oral and written informed consent forms. In a context of courtesy and respect, the respondents were asked whether they were ready to participate voluntarily. All subjects explained about the aim and benefits of the current study. Then, they approved to participate in the interview. Confidentiality was maintained, and then research participants were identified using the codes instead of their names. All notes and audiotapes related to the perspectives of the participants were taken.

## Results

To present the results of this study for identifying the barriers to mental health service utilization, three main themes emerged after data analysis, which were (a) fear of stigmatization and lack of awareness of mental health, (b) sociocultural barriers, and (c) financial and geographical difficulties to mental health services, which were used as the fundamental barriers to mental health care provided among the patients seeking health treatments and therapies at Kabutare Hospital (Table 1).

**Table 1:** An overview of the theme, categories, and their subcategories.

**Themes**	**Subthemes**
Fear of stigmatization and lack of awareness of mental health	•Lack of awareness of the professional mental health services •Stigmatization and their consequences •Negative attitudes of society toward mental disorders
Sociocultural barriers	•Societal beliefs in traditional healers and prayers •Lack of patient care and relapse
Financial and geographical accessibility barriers	•High cost of mental health services and health insurance •Geographical accessibility to mental health services •Financial concerns toward psychotropic medications

The results from this study are presented with an initial summary of the themes followed by the subthemes corresponding to each main theme. All results are illustrated with quotations from the interviewed participants, presented in italics. The subthemes were also displayed in italics for differentiating them from the main themes.

### Fear of Stigmatization and Awareness of Mental Disorders

In the physical barriers to use mental health services, the researchers found two main barriers emerging, namely, (a) lack of awareness of mental disorders and (b) fear of stigmatization.

#### Lack of Awareness of the Professional Mental Health Services

Poor awareness of mental disorders is the barrier that hampers the access and use of mental health services in most people with mental disorders. For instance, during the interviews, 8 out of 10 interviewed patients present a problem of poor knowledge about their mental health conditions. Some patients with mental disorders seek healthcare supports lately because different family members fear to bring the patients at the health facility since they have poor awareness on the causes of mental disorders and also dispel the myths around the predictors of these disorders and their psychotherapies or therapy options. The family members of the patients with mental disorders also think that the health facility have insufficient healthcare services namely healthcare professionals and medications that may lessen the severity of the mental disorders. The interviewee indicated that lack of awareness is an important barrier to receiving mental health care:

“*I only knew other people's disease like malaria and I just used to look at people who are sick. I didn't have any thoughts about mental health conditions. I am just worried about feeding my family and seeing to it that their needs were met. This was because I knew nothing about these mental disorders,”* (Male, 39 years).

Participants seeking health care at the recruited hospital also showed they were poorly knowledgeable about the availability and accessibility of mental health interventions and treatments. They were uncertain about which suitable settings or health facility they may seek for mental health services, and these obstacles weaken their willingness to seek for mental health services. Another interviewed patient also expressed that lack of awareness was the barrier to accessing mental health.

“*I was not aware of this place and I felt the illness need spiritual attention and we delayed coming so the situation got worse and worse,”* (female, 49 years).

Seeking mental health from traditional healers was importantly discussed as a common phenomenon among the interviewed patients of this study. For many participants, they believe that mental disorders should be treated effectively by traditional and faith healers than professionals from the hospital. This is because they think that whatever happens to a person which affects the nervous system is related to the devil and witchcraft. Besides, exorcism to get health improvement was stated as another means due to their belief in evil spirits whom they believe that they have power to chase away. Additionally, mental health patients expressed that they occasionally seek health intervention from church prayers where they believe that engaging in prayer for a long time helps them to get a recovery. Thus, the patients seek mental health care at the medical or psychiatric setting after failing to get an improvement through traditional healers and prayers. The interviewed patients expressed that poor knowledge of the mental health services that are available at the hospital limits them to seek care for mental health.

“*When I started having the symptoms of mental disorder, I did not know where to seek care for mental disorders and my family was unaware. My family relatives brought me to traditional healers for seeking health support. As I got no health improvement, they also brought to the prayers. Unfortunately, the symptoms continued being more severe until when I was brought here” (male aged 29 years)*.

The community has poor awareness of how mental health services play a great role in mental well-being improvement. The community members consider one patient with mental disorder to have been affected by the devil and then start harassing the patient by calling him non-appropriate names.

“*When my mental disorder started, my father was not aware of my disorder. Then, I was taken to a faith healer for seeking support. They refused to bring me here they took me to some prayers and other places before we were directed to come to this hospital*” (female, 22 years).

However, for patients with mental disorders, they indicated that they knew their mental disorder, symptoms, and etiologies. An interviewee expressed:

“*A disorder that results in changing behaviors of someone such that he undresses in public and start having violent behaviors, isolating himself from others, talking to himself, and eating unhealthy meals*.” (male, 30 years).

Patients with mental disorders demonstrated that mental disorders refer to the change in behaviors from what they used to do before and what they do presently as unusual behaviors, which then may be taken as a kind of mental health disorder. In this case, when the community starts harassing them, they develop disrupted behaviors and feelings such as shame and stigma. From the quotes taken from the respondents including unhealthy eating patterns (such as snacking, meal skipping, inconvenience, and eat poor food) and being aggressive to others with no significant reason, the subject expressed these in the following perspectives:

“*A disorder that results in changing behaviors of someone such that he undresses in public and start having violent behaviors, isolating himself from others, talking to himself, and eating unhealthy meals (male, 30 years)*.

#### Fear of Stigmatization

All participants indicated that they self-stigmatized well, and eight of the participants also indicated that they experienced social stigma related to their mental disorder. Some of the participants expressed that they often possess the stigma of attaching mental disorder to the community members and some of their family members, so they deny to bring the patients to the hospital so that no one could know that they have a patient with mental health condition. For them, the stigma caused them to become marginalized, resulting in a delay in receiving mental health services and refusing to be taken to the hospital. The patients who were interviewed in this study indicated that the community members often have negative attitudes and perceptions toward their mental health disorders. For the interviewees, the community lacks empathy and this negatively impacts the families of these patients and causes the patients with mental health conditions to experience psychosocial issues including feeling frustrated, shameful, and socially neglected.

#### Negative Attitudes of Society Toward Mental Disorders

Participants responded that being mentally ill was stigmatizing and this kind of stigmatization hinders the patients from receiving mental healthcare services. They also indicated that the patients with mental disorder developed self and social stigma from the community and family in which the patient resides. The results revealed that self-stigma occurred when the patients were aware of their mental disorder which then resulted in development of hopelessness as well as poor adherence to mental health services. The participants generally described negative attitudes of members of their communities who used stigmatized words or names due to their mental health conditions. Research participants expressed that patients having a mental disorder was regarded as “BAD” by community members, regardless of the diagnosis. They also expressed social stigma and social exclusion that caused them to marginalize themselves and drop out from social identification or potential social groups:

“*After getting ill [epileptic case] my classmates started isolating me because they were thinking that I could cause them disorders. Due to this kind of exclusion, they started calling me another stigmatizing name like SEBICURI. This social exclusion and marginalization caused me to decide to drop out of my school.” (male, 24 years)*

The other barrier that was found was the loss of opportunities like interpersonal relationships and loss of trust from their colleagues and other opportunities that made them socialize with others. These influenced their stigma and then fell neglected by the community. The subjects expressed their loss of important opportunities that might contribute to their development. An interview expressed her perspective in the following words:

“*My friends isolate me from different activities like in sport; working and interacting with were stopped, because of my mental disorder,”* (female 22 years).

Patients with mental disorders showed problem of loss of their family duties due to their mental disorder and their symptomatology. For the married men, their spouses neglect them and marginalize them. The patient aged 39 years indicated the barriers faced while seeking mental health services:

“*For the standing of the disorders I lost everything, job, friend and even my wife live me because at the starting I was very aggressive and it became a big problem for me. You can suffer from other diseases like malaria or another one but suffering mental disorder is not easy. You lost everything.”* (male, 47 years).

#### Self-Stigmatization

Self-stigma is also a big issue for mentally ill patients. Almost all the interviewees who took part in this study expressed that they developed negative attitudes and behaviors due to being marginalized and stigmatized by their families and communities. One of the research subjects expressed he still lives with self-stigma which persisted long after his first contact with mental health providers:

“*I lost many opportunities due to suffering from a mental disorder, I lost hope for the future, and I was isolating myself because I don't want to meet with any person to avoid, they can laugh at me”* (male, 22 years).

When the psychiatric patients were interviewed, they also expressed several mental health issues characterized by loss of hope, fear of stigmatization, self-neglect, and living in poor health conditions due to the negative attitudes of the community toward their mental health conditions. She expressed her challenges in the following perspectives:

“*It is not easy to live with this mental disorder, because people around you start to have fear for me, and I can't give an idea or advice and accepted because they used to say that it is for foul people or mad person. They can't recognize that people suffering from a mental disorder can have recovered, it not easy and I sometimes hate myself.” (Female, 27 years)*.

Some participants of this study expressed in their perspectives that their lower accessibility to the services related to mental health disorders was caused by shame which pushed them to hide their mental health conditions to themselves. The patients sometimes delay to take mental health services and personally stop seeking the mental health services at the hospital. For them, having mental disorders is shameful. A participant expressed:

“*I live the life of shame because of being mentally disordered, due to the negative perceptions of other toward my disorder, I sometimes decide not to seek therapies at the hospital and if went to look for help I hide so no person can see me in consultation at the hospital, but I notice no improvement and then decide to live with my disorder forever.”* (male, 49 years).

In their perspectives, the patients who were interviewed expressed that they had fear of implications and disclosing mental health disorders to other community members and they described that their fear, in the previous time, caused them to hide the psychological distress that they were experiencing in their daily lives. Participants also said that fear of social stigma had been a barrier to their access to mental health therapies. The patient expressed:

“*I feel that if they knew about me.knew about my depression, their opinions would reject me because I'm like that, and they feel I'm abnormal. I just wouldn't tell them that I had mental disorder [depression] because they would say, ‘Get away from me! You’re mentally ill”* (female, 33 years).

Patients with mental disorders experience negative attitudes of community toward their disorders, and the society considers them as being unable to do anything. The results from the interviews evidenced that the community members perceived mental disorders to be caused by possession of evil spirits. Another patient expressed:

“*It stopped me from looking the help [utilizing mental health services], like I said, if they have a bad opinion of me, I might as well keep it the same, it doesn't matter. If they don't care, why should I? Didn't want to help myself, didn't want to get help because if they thought of me like that, maybe the person I was going to see was going to think of me like that*.” (female, 33 years).

Patients expressed that their poor seeking of mental health services occurs due to the negative attitudes of the community toward their mental disorders. They become ashamed of being mentally ill because the community perceived them as people who have strange behaviors. Another participant said:

“*Other people look at it and say 'Well, she's going there, she's obviously like really bad', like, why is she let out because she has to go to psych services, isn't that where all the crazy people go? She's crazy too. She has a lot of problems because she has to go there'. So, this became a barrier to us for not going to look for mental health service. Even if we go for seeking healthcare we keep it as a secret so that no person can know it. This keeps us not to become ashamed of our disorder”* (male, 22 years).

The occurrence of gossiping social networks was raised as an issue that lessened the utilization of mental health services. The interviewed patients expressed how quickly information can travel within socially proximate rural areas or rural settings where this gossiping network is due to social influences. The results indicated that the community gossip was a significant issue for these research subjects to the extent that they believed that their progress toward recovery had been negatively affected by it. In their perspectives, the interviews expressed that the young people in their community had not contended with this health issue. A participant expressed her perspectives, saying:

“*Just the way people talk about you in this town. It's disgusting. Yeah, just the gossip and everything. I know I really shouldn't care what other people think and everything but it gets a bit hard when everyone's at school, or everyone in your group of friends starts talking about you. It's like you've got nowhere to go so you just stay home.That's why living in the city is better than rural since you can't know everyone and you can get a second chance. If everyone knows, you can't have one and people are forced to move out of town before”* (female, 25 years).

### Sociocultural Barriers to Mental Health Service Utilization

#### Societal Beliefs in Traditional and Faith Healers

For traditional healing of the patients with mental disorders, the traditional healers are often from the families with historical background of providing traditional healing for several patients with various health problems in their respective societies, as the knowledge and experience to treat mental disorders. This kind of medical practice is taken as the family acquiescence that is transferred from one family to another. Within this system of traditional healing, traditional healers mostly use the combination of various herbs and the “secret knowledge,” and in some circumstances, rituals in the community are used for helping the patients. These traditional healers are respected in their communities and societies, and through this respect, they are involved not only in treating the disorders but also in solving disputes and other psychosocial problems within their communities. In addition to that, religious healers particularly the Protestants also live in the community and share sociocultural beliefs with the community members. However, in this system of healing patients with mental disorders, the treatments used include religious texts that are recited and written on paper or special materials as well as preparing solutions; the solutions mostly used involved washing the body, drinking, and both drinking and washing hands. The religious healers are considered as people whose background is religion, and they are mostly respected by people in their society. Most of the time, they intervene in sociocultural events including naming ceremonies, burial services, and teaching and preaching religion. They are mostly in every community and are accepted by the communities. Based on the perspectives from the participants in this study, they are taken to faith healers and prayers to avoid social stigma and marginalization from their community members. The perspectives of the interviewed patients demonstrated that the community members and family relatives of the patients are not aware of their mental disorders because they think that these mental disorders are the results of external power such as evil spirits, demons, witchcraft, and foul wind. This is why they believe that these kinds of mental health conditions are not effectively curable by biomedicine or modern medications, but that the only effective treatment is the one provided by traditional and faith healers. As a result, seeking mental health services in medical settings are considered as the last resort when no other actions taken improved their mental well-being. Based on the abovementioned reasons, patients indicated that to avoid social stigma, they actually seek health care from traditional and faith healers who are accepted by their communities:

“*Most mental illnesses are the results of evil spirits, demons, and devils. These types of mental disorders are difficult to be treated by modern treatment. It involves fighting with the spirit, with the demons, with the devils. Not every healer can do it effectively. For these illnesses, the health care provider from the hospital can't afford effective treatment for mental disorders. As a result, receiving treatment from there may make patient with mental disorders worse”* (male, 50 years).

The utilization of the religious system in treating people with mental disorders depends on the religious background and families of patients with such mental conditions. Concerning the costs for the treatment, there is no specific or standard cost of the treatments from traditional and faith healers but the costs of mental health services may be changed depending on the length of the treatments and major causes of the diseases. Most of the time, the payments are provided through the agreement between the patient and the healer. The participant expressed:

“*My mother paid a huge sum of money to the traditional healers. In our rural area and another in the western province, she was asked to pay half of the cost of the treatment and when I get well”* (female, 27 years).

Although accessibility to traditional and religious treatments was mostly common among the participants for this study, they took it as another difficult problem since they spent much. These systems of treating the patients with mental disorders have become a lucrative business undertaking according to the participants of this study. Some patients expressed that these systems of treating them led to fake healing characterized by charging patients to pay high costs and sometimes some of their properties for healing:

“*Now the problem is many people pretend to be traditional healers when they cannot do anything, just to take peoples' money. So, who stays believes they are going to suffer because they charge a lot now traditional healing is costlier than biomedical treatment?”* (male, 39 years).

Certainly, the traditional and religious use way of treating the patients with mental disorders challenges the patients and this might lead to health burdens and risk the life of patients with mental health conditions. This is because these treatments are characterized by beating and chaining the patients seeking the intervention. A participant expressed how he was treated by the traditional healer:

“*I could not continue to stay to take treatment. Because here you see they chain some people. Also, you see people been beating during the treatment. I do not want to see that,”* (female, 27 years).

While the family members saw this as the first solution due to the “supernatural forces” nature of mental health conditions, the medical staff complained that consultation with traditional healers usually interfered with the treatment pathway of a patient. Mental health conditions are chronic conditions; they always need to be watched and managed. Because of this, most families also ran out of patience when looking after their patients. This caused them to seek alternative treatments that affected the compliance with medication and care. Traditional healing and religion contributed to the low consultation for mental health services because the same participant believed that the diseases come from the devil. Results showed that the patients with mental illnesses ignored and had a lack of awareness about mental disorders which led them to consult with traditional healers instead of coming to mental health service care.

#### Lack of Patient Care and Relapse

Lack of patient care and relapse were mentioned as the barriers to utilization of mental health services. We found that if more care was given to the patients both at the facility and when they are discharged, they would have had fewer relapse cases. It was found that family members treated the hospital as a dumping ground for them to leave and forget about their family members. It was reported that sometimes the medication is strong enough to cause some side effects. Due to this, they may not have sufficient food to help cope with the side effects of the prescribed drugs. They also mentioned that when the patients were treated and went back to their homes, there was no awareness and they ended up getting involved in the activities that got them sick in the first place, especially for substance abuse-related conditions. The participant expressed his barrier in the following perspectives:

“*A lot of mental disorders in young adults or adolescents result from the use of drug abuse like cannabis, heroin, alcohol which are being widely used in the community around the country. So, you see if somehow they are recovered and move to the same area or community they can do it again hence relapse, so we need more awareness”* (male 39 years).

Concerning relapse, the following section describes the financial and geographical limitations.

### Financial and Geographical Limitations

Financial barriers to utilization of mental health services included poor financial means which weakens the accessibility to the services. In this economic implication, the research findings reported the themes related to the barriers including travel costs, high costs of the mental health services, and poor quality of the services provided.

#### High Cost of Mental Health Services and Health Insurance

Appropriate accessibility to healthcare intervention is described as the availability of healthcare services to be within reasonable reach to those who need healthcare intervention; affordability is described as the ability to pay for mental health services without financial challenges; and acceptability is described as the willingness of patients to seek for mental health services. The participants also expressed that although they have health insurance to be at the health facility, they sometimes lack the prescribed medications so they purchase them at private pharmacies where the medications are very expensive. In that way, they suggested that psychiatric medications should be provided for free or the costs the hospital provided be lowered:

“*It's harder to get around if you don't have money at all and because I live in a rural area if hard to find a car, it requires to take a motorcycle and its very expensive, so sometimes we decide to stay at home transportation is yeah, a big problem here”* (male aged 22 years).

Another participant expressed:

“*When we used the health insurance at the hospital, we become encouraged to access mental health services, but sometimes, we lack medications at the hospital and the health care providers transfer us to the private pharmacies for buying medications. In these pharmacies, the health insurance does not cover prescribed medications and the drugs are very expensive. As this increases the burdens for us and our families, we become discouraged to received medications at the hospital and when the complications later come we also go to the hospital or not. For me, if all prescribed medications are available at the hospital and the side effects decrease accessibility to mental health services may increase”* (female, 45 years).

#### Geographical Accessibility and Affordability Barriers to Mental Health Services

The subjects indicated that the geographical accessibility to mental health service utilization is a tangible barrier to the utilization of mental health interventions in the recruited medical setting. It was found that lack of transportation is a serious issue for people and that all interviewed participants presented this as a barrier for not consulting with mental health services in the health facility because of poverty, so that people decide to look for traditional medicines near them that can help them. Another subject raised is the problem of difficulty in trajectory and accommodation because the same health facilities are far so it became a big challenge to seek help. A female participant expressed the barrier to mental health use in the following perspective:

“*My family lives far from the city, so when it's time to go back to take medication, it became a big problem to find the money for transport, accommodation, food and I can find anyone to go with me and sometimes,”* (male, 25 years).

#### Financial Concerns Toward Psychotropic Medications

In the medical setting, it was found that patients seeking mental health services cannot utilize them due to not being healthily insured since their health insurance did not cover all the expenses for mental health medications and therapies that could promote their mental well-being. The patients with no extra money in their pockets faced the burden of paying the expensive psychotropic drugs. These experiences were found to cause the delay to take medications and loss of trust for the psychotropic drug effectiveness. A subject aged 39 expressed this barrier to mental health care in the following. Also, most of the participants described the inability to buy the prescribed medication due to lack of money and that their health insurance that does not cover the prescribed medications.

“*Medication is very expensive, so my health insurance cannot cover all money I am charged. This caused not to take the prescribed medications. It is too difficult to recover effectively and efficiently,”* (male, 50 years).

The patients suggested that services should be more affordable or totally free, particularly for the low-income group, to reduce the burden of illness on their families. The results of this study indicated that the lack of money to pay was the barrier for the patients from the poorest families and who had no health insurance to cover the medications. One patient expressed:

“*I think to meet up with these expenses is not easy, the problem here is that there is no specific amount. Whatever you are asked to pay you have to do like today I was not expecting to pay that lot of money for this drug,”* (female, 27 years).

Although patients with mental disorders have access to different health care services for their recovery, the link between the causes of the mental disorders and the current treatment pathways changes along the treatment continuum, and such a change in the treatment patterns was associated with the satisfaction level in the aspect of seeking health therapies from traditional healers and faith healers. These services hamper the accessibility to suitable mental health services provided at the health institutions and health facilities. Decisions to receive treatment from faith and traditional healers are made because the patients consider medications from the hospital as futile with respect to their sufferings in behavior, thinking, and emotions.

## Discussion

The current study is the first to be conducted among the mentally ill patients in Rwanda, particularly at Kabutare Hospital in Southern Province, Rwanda. Its primordial purpose was to explore the barriers to mental healthcare service utilization among the patients with mental disorders. Results revealed that there were various barriers to access and utilize mental health services. Three main barriers were explored: (a) fear of stigmatization and poor awareness of mental health, (b) sociocultural barriers and financial strains, and (c) geographical limits. These results are in line with the preceding studies that documented a range of barriers to seeking mental health services in LMICs (Evans et al., 2015; Benti et al., 2016).

Concurrent with prior studies that indicated that the negative attitudes of communities toward mentally disordered patients are the major barrier which caused mentally ill patients not to seek mental health services and to develop poor adherence to mental health interventions (Power, 2002; WHO, 2002; Evans et al., 2015), deviant behaviors and negative attitudes toward mental disorders were revealed to hinder the access to and utilization of mental health services. The abovementioned way of labeling people causes stigmatization which affects their healthcare seeking for their mental disorders, as it is related to shameful and degrading acts. These findings were relevant to the previous studies that indicated that patients with mental disorders encountered the stigma that affects the accessibility to mental health services (Verhaeghe et al., 2008; Luborsky and Rubinstein, 2011). Concurrent with former studies that documented that people with mental illnesses were poorer in comparison with patients without mental disorders (Verhaeghe and Bruynooghe, 2007; Sorketti et al., 2012; Staiger et al., 2017), our results revealed social stigma and lack of empathy among the patients with mental disorders. These barriers result in feeling neglected and discriminated and shame for the patients with mental disorders, which hamper their seeking for mental health services at the health setting. In addition to that, financial strain and high costs for mental health services particularly psychotropic medication limit the patients to receive mental health care at the hospital. These results are in line with the erstwhile studies (Braun and Clarke, 2006; Nowell et al., 2017).

Our results revealed that fear of stigmatization and low level of awareness of mental disorders are the most common barriers that negatively affect the possibility to seek mental health services. They revealed that social stigma and shame in seeking health care from the hospital were the main constraints that hinder the utilization of mental services; however, these barriers are also important environmental factors in the health belief model (HBM). These strains cause the patients to withdraw from seeking any mental health support from the hospital (Byrow et al., 2007; Abdelgadir, 2012; Jack-Ide and Uys, 2013; Roberts et al., 2018). The results demonstrated that their family relatives played an important role in their effective and efficient health intervention; however, they encounter the problem of poor awareness of mental disorders. Our results are in a similar vein with prior studies (Molodynski et al., 2017; Staiger et al., 2017). Further, concurrent with the erstwhile studies conducted in sub-Saharan African countries such as Tanzania, South Sudan, Uganda, Kenya, Ethiopia, and Ghana which documented that patients with mental disorders often prefer to seek relief for their mental disorders from traditional healers and prayers [Abbo, 2011; Abdelgadir, 2012; African Technology Policy Studies (ATPS), 2013; Mbwayo and Ndetei, 2013; Ae-ngibise et al., 2014; Uwakwe and Otakpor, 2014], our results revealed that the beliefs and attitudes of the patients seeking alternatives to mental healthcare services were important barriers to the utilization of the services from the hospital, particularly as consulting with traditional and faith healers was one the most common alternatives to mental health services. Normally, the patients with mental disorders seek mental health services at the hospital at a later stage, after religious and traditional healers have failed to cure their mental illnesses. This means that seeking health care at the traditional and faith healers were remedy for most respondents. Their perspectives revealed that seeking healthcare services was their last preference for them after the healthcare services from the traditional and faith healers fail to treat mental health conditions of the respondents. However, some of the research conducted in some sub-Saharan African countries like Kenya and Uganda has yielded contradictory findings that showed that traditional and faith healers prominently contribute to mental health improvement. Indeed, the only patients with mental disorders who are often brought to the hospital are those who do not present health improvement after being treated by traditional and faith healers (Henderson et al., 2013; Jack-Ide and Uys, 2013; Rugema et al., 2015; Ali and Agyapong, 2020).

Preceding studies indicated that geographical accessibility limits especially in sub-Saharan African countries are an important barrier that hinders health-seeking for patients with mental disorders since this barrier increases privation and delay for seeking mental health services. These findings are also in the same vein with the previous studies conducted in Nigeria, Ethiopia, and South Africa where different systems of treating patients are applied (Abdulmalik and Sale, 2012; Jack-Ide and Uys, 2013; Ambikile and Iseselo, 2017; Musyimi et al., 2017). Additionally, financial strains limit their access to mental health services since the cost of some medications such as antipsychotic drugs is too much for them to shoulder on a long term. This suggests the existence of a huge financial difficulty on carers of psychiatric patients due to the cost of even the cheapest antipsychotic drugs. Additional barriers, including seeking mental health services from traditional and faith healers, the lack of need for treatments, or the high cost of medications, were described as barriers to mental health policy implementation in the hospital. The results of this study were relevant to the previous studies carried out in Tanzania which reported how traditional healers have charged too much for their services for treating mental disorders (Muela et al., 2012; Mwansisya et al., 2015; Rugema et al., 2015; Ali and Agyapong, 2020; Blixen et al., 2020; Iseselo and Ambikile, 2020).

### Strengths and Limitations

This study had several strengths that made the study valid. First, the primordial strength of the current study is that it brought novel knowledge based on the perspectives from the participants since there was no preceding study conducted among the mentally ill patients to explore the barriers to receiving mental health interventions. Second, the data reflected real-life practices which presented the barriers encountered by the patients with mental health conditions. However, some limitations were encountered in this study. First, the study was limited to lack of interview with psychiatric nurses, pharmacists, psychiatrists, psychologists, and social workers from the health facility who are involved in the provision of health services. Their involvement in the study should give the researchers different perspectives about the barriers to mental health utilization among patients with mental disorders. Second, the study was limited to the study area because it was conducted in a sole district hospital.

## Conclusion

To conclude, this study found several barriers to mental health utilization that affect the ability of the patients with mental disorders. The most prominent barriers to a low access and utilization of mental health services at the hospital were fear of stigmatization and awareness of mental disorders, strain of financial supports and limits to geographical accessibility, and sociocultural limits. Seeking help from traditional healers and faith healers and lack of empathy limit patients from receiving mental health services at the hospital. Based on the results of this study, the commitment of policymakers which may play a great role by designing policies targeting health education for addressing the barriers is recommended to increasing the magnitude of using mental health services. Further, researches designing health communication campaigns with the purpose of decreasing the barriers to utilization of mental healthcare services are recommended. A research on the factors associated with low access to mental health care is recommended. For instance, the Ministry of Health, in Rwanda, has a clear health strategy on training and motivation on mental health providers as well as integration of mental health services into primary healthcare. However, we think that media could play a crucial role in increasing the level of awareness of mental health conditions and their treatability. Second, a longitudinal study to investigate the influences of individual characteristics or sociodemographic characteristics and social context variables on utilization of mental health services over a certain period is recommended.

## Data Availability Statement

The raw data supporting the conclusions of this article will be made available by the authors, without undue reservation.

## Consent for Publication

Consent for publication was obtained from the participants. The participants approved and provided their undersigned informed consent for publication of their perspectives.

## Ethics Statement

The studies involving human participants were reviewed and approved by Institutional Review Board, College of Medicine and Health Sciences, at the University of Rwanda. The patients/participants provided their written informed consent to participate in this study.

## Author's Note

Oliviette is a graduate student in an undergraduate program in Clinical Psychology at the University of Rwanda. She is currently working in SOS Rwanda for providing health care to children. Emmanuel held a Master's Degree in Public Health and Bachelor's Degree in Clinical Psychology at the University of Rwanda. He is the Program Manager of Rwanda Resilience and Grounding Organization (RRGO). He is a District Manager of Prison Fellowship Rwanda. He is a professional trainer of Community Resilience Model Skills and Laughter Yoga approaches.

## Author Contributions

OM and EB made substantial contributions to the conceptualization, study design, data acquisition, research administration, data analysis and interpretation of data, and study visualization. EB drafted the manuscript and revised it critically for important intellectual content. All authors read and edited the manuscript, agreed to be accountable for all aspects for this research in ensuring that questions related to the accuracy and integrity are appropriately addressed, and approved the final manuscript.

## Conflict of Interest

The authors declare that the research was conducted in the absence of any commercial or financial relationships that could be construed as a potential conflict of interest.
