# Supplementary material for: Exploring Barriers to Mental Health Services Utilization at Kabutare District Hospital of Rwanda: Perspectives From Patients
Source: Front Psychol. 2021 Mar 22;12:638377. doi: 10.3389/fpsyg.2021.638377 (PMC8019821; doi:10.3389/fpsyg.2021.638377)
Supplement: Supplementary file 1 [file Data_Sheet_1.PDF]

Interview guide

To address to the research objectives, the researches designed the interview guide based on the prior and pertinent studies conducted especially in sub-Saharan African countries. The following questions were asked to the participants, however, further questions were asked depending on the perspectives from the participants:

1. From your experience as a patient under mental health services at this hospital, what do you do when you realize that you suffer from mental disorders?
2. From your experiences in seeking healthcare services, what are personal barriers have you encountered as a reason for not seeking health care at health facility?
3. What are the social influences or bottlenecks that impede integration of mental health care at the hospital?
4. What are cultural and religious influences that encumber your utilization of mental health care services from this hospital? When you do not seek healthcare services at the hospital, where else do you seek it?
5. What are environmental challenges do you face that hinder you to seek mental health care services from the hospital?
6. What are your perceptions and attitudes of your family and community members toward your mental disorders?
7. What are financial barriers do you face that hinder your reception of mental health care services from the hospital?
8. If you have any other barrier to mental health services utilization, your perspectives are welcomed.

Thank you for your participation!
